# Dosimetric assessment of brass mesh bolus for postmastectomy photon radiotherapy

**DOI:** 10.1120/jacmp.v17i6.6221

**Published:** 2016-07-25

**Authors:** Ryan Manger, Adam Paxton, Laura Cerviño

**Affiliations:** ^1^ Department of Radiation Medicine and Applied Sciences University of California San Diego La Jolla CA USA; ^2^ Department of Radiation Oncology University of Utah Salt Lake City UT USA

**Keywords:** bolus, breast, Monte Carlo, activation, neutron

## Abstract

Brass mesh bolus has been shown to be an acceptable substitute for tissue‐equivalent bolus to increase superficial dose for chest wall tangent photon radiotherapy. This work investigated the increase in surface dose, the change in the dose at depth, and the safety implications of higher energy photon beams when using brass mesh bolus for postmastectomy chest wall radiotherapy. A photon tangent plan was delivered to a thorax phantom, and the superficial dose ranged from 40%–72% of prescription dose with no bolus. The surface dose increased to 75%–110% of prescription dose with brass mesh bolus and 85%–109% of prescription dose with tissue‐equivalent bolus. It was also found that the dose at depth when using brass mesh bolus is comparable to that measured with no bolus for en face and oblique incidence. Monte Carlo calculations were used to assess the photoneutron production from brass mesh bolus used with 15 MV and 24 MV photon beams. The effective dose from photoneutrons was approximated and found to be relatively small, yet not negligible. Activation products generated by these photoneutrons, the surface dose rate due to the activation products, and the half‐life of the activation products were also considered in this work. The authors conclude that brass mesh bolus is a reasonable alternative to tissue‐equivalent bolus, and it may be used with high‐energy beam; but one should be aware of the potential increased effective dose to staff and patients due to the activation products produced by photoneutrons.

PACS number(s): 87.53.Kn, 87.55.K

## I. INTRODUCTION

Postmastecomy chest wall radiotherapy (PMRT) is a common treatment modality for high‐risk breast cancer patients following mastectomy because it has been shown to improve local control and survival.^1^, ^2^, ^3^ The RTOG breast atlas^(4)^ defines the chest‐wall target volume as extending cranially to the caudal border of the clavicle head, caudally to the loss of computed‐tomography (CT) apparent contralateral breast, laterally to the mid‐axillary line, medially to the sternal‐rib junction, posteriorly to the rib‐pleural interface, and anteriorly to the skin. Several radiotherapy methods may be used to cover this target volume, but the technique of interest in this work is the most prevalent method, the tangent photon beam arrangement.

When treating the chest wall with tangent, high‐energy photon beams, the anterior aspect of the chest‐wall target, which extends to the skin, may be inadequately covered due to the skin sparing effect. In a retrospective study of 61 inflammatory breast cancer patients, Thomas et al.^(5)^ found the most common site of failure was in the chest wall, and, specifically, failure was most common in patients who did not achieve brisk erythema or moist desquamation. Hence, to ensure sufficient superficial coverage, tissue‐equivalent bolus is commonly used for a portion of the treatment until moderate to brisk erythema is observed. The use of tissue‐equivalent bolus has a few unwanted consequences. One of these is the lack of conformity to the chest wall, which may result in decreased surface dose. It has been shown that the surface dose may decrease by as much as 10% for air gaps up to 10 mm.^(6)^ Another issue with tissue‐equivalent bolus is the requirement of two treatment plans — one for bolus and the other for no bolus — due the attenuation differences in the absence and presence of bolus.

**Figure 1:**
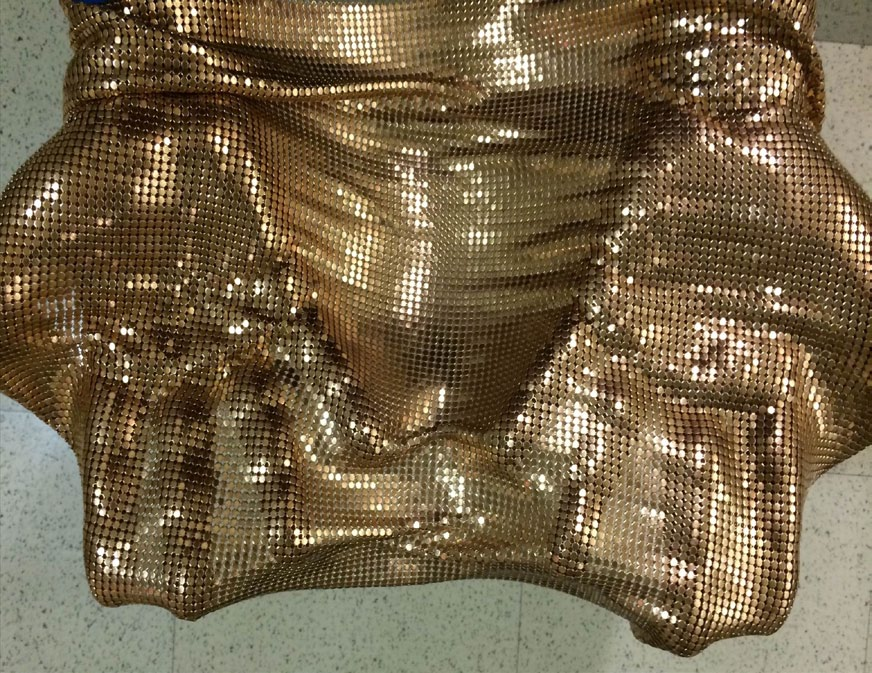
Brass mesh bolus.

An alternative to tissue‐equivalent bolus that has been used by other institutions is brass mesh bolus (Fig. 1). The brass mesh bolus is constructed similar to riveted chain mail, where brass rivets are linked together to form a mesh. One of the reasons for using brass bolus instead of tissue‐equivalent bolus is the improved conformity to the chest wall. Healy et al.^(7)^ showed that using brass mesh bolus resulted in surface doses between 81% and 122% of the prescribed dose in 16 female patients receiving PMRT. Surface doses were estimated using thermoluminescent dosimeters (TLDs). Another benefit of using brass mesh bolus is the reduced impact on the dose at depth compared to tissue‐equivalent bolus.^(8)^

The goals of this work are to determine the effects of brass mesh bolus on dose at depth, the effects of brass mesh bolus on the dose near the surface in tangential beams, and the safety implications of using brass mesh bolus with higher energy photon beams.

## II. MATERIALS AND METHODS

### A. Percent depth dose (PDD)

The central axis (CAX) PDDs for $15\;\times \;15\;{\text{cm}}^{2}$, 6 MV and 15 MV photon beams were measured using an Advanced Markus ionization chamber (PTW, Freiburg, Germany) at several depths ranging from 0 cm to 10 cm in a plastic water‐equivalent phantom (CIRS, Inc., Norfolk, VA). The phantom was set at 100 cm source‐to‐surface distance (SSD) on a Varian Clinac iX Linear accelerator (Varian Medical Systems, Palo Alto, CA). These measurements were repeated with a 0.5 cm thick layer of tissue‐equivalent Superflab bolus (Q‐Fix, Avondale, PA) placed on top of the phantom, and then with a layer of brass mesh bolus (Whiting & Davis, Attleboro Falls, MA) placed on top of the phantom.

### B. Dose‐at‐depth with tangential beams

Since en face PDD measurements are not representative of actual treatment geometries, the dose at depth for a beam entering at an angle of 45° off‐normal was considered. A PTW Semiflex ionization chamber (TN31010) was placed at the central axis in a solid water phantom at a depth of 10 cm, 100 cm SAD. Measurements were acquired for a $15\;\times \;15\;{\text{cm}}^{2}$ 6 MV photon beam with en face incidence and 45° off‐normal. The measurements were repeated with a 0.5 cm thick layer of tissue‐equivalent Superflab bolus placed on top of the phantom, and then with a layer of brass mesh bolus placed on top of the phantom. The ratios to doses with no bolus were compared to determine the effect of the bolus material at depth with the various bolus materials in place.

### C. Surface dose on a thorax phantom with tangential beams

**Figure 2:**
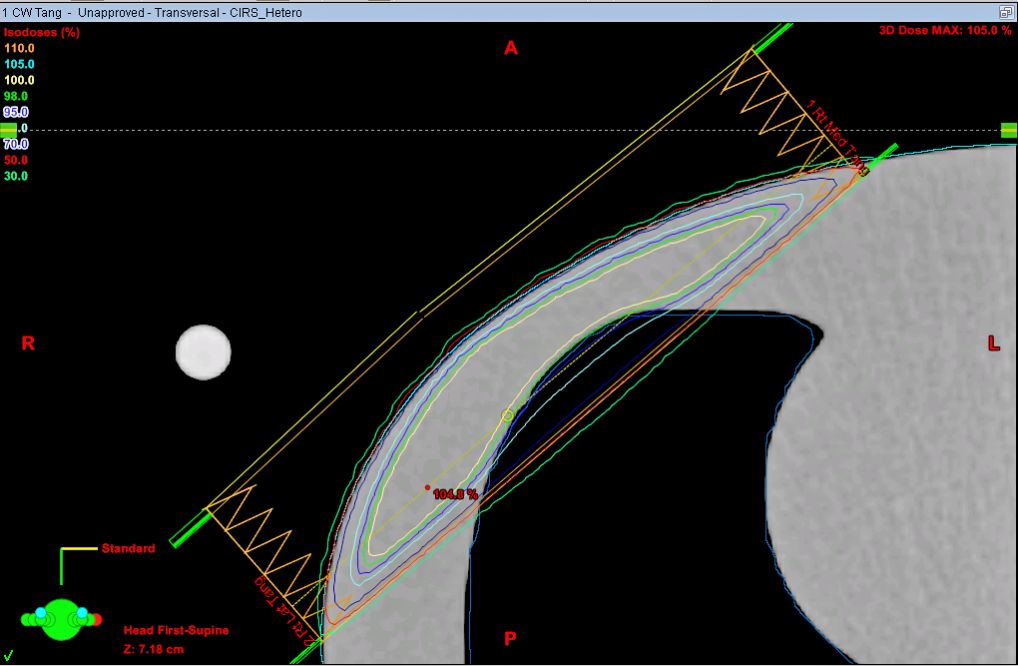
An axial slice of the 6 MV photon tangent chest‐wall plan for the CIRS heterogeneous IMRT thorax phantom.

**Figure 3:**
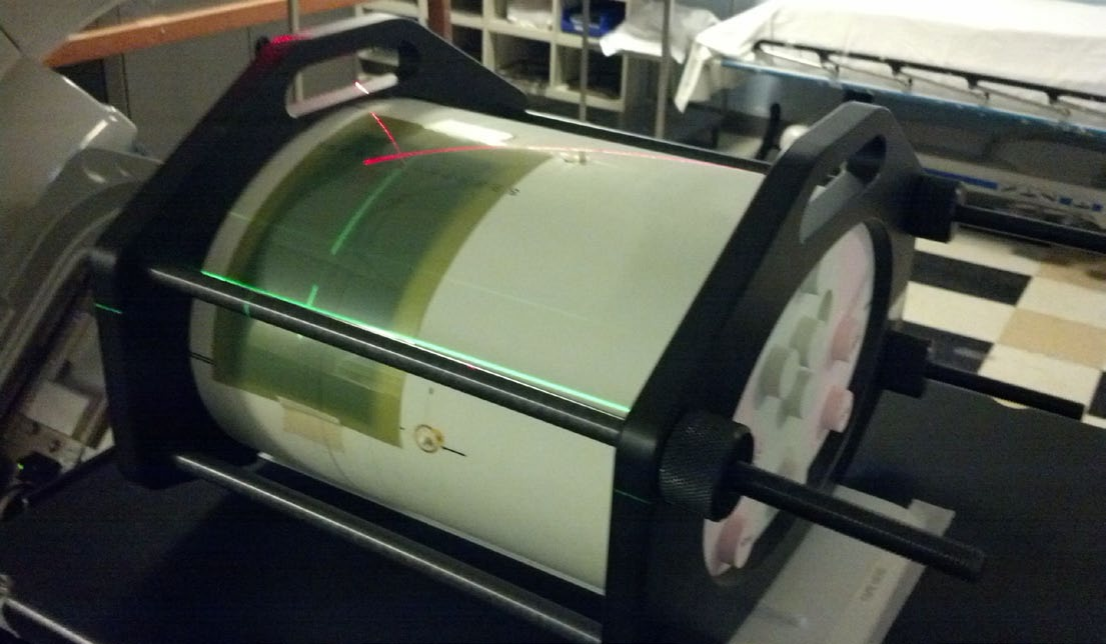
Experimental setup for measuring the surface dose on the IMRT thorax phantom using Gafchromic EBT3 film.

**Figure 4:**
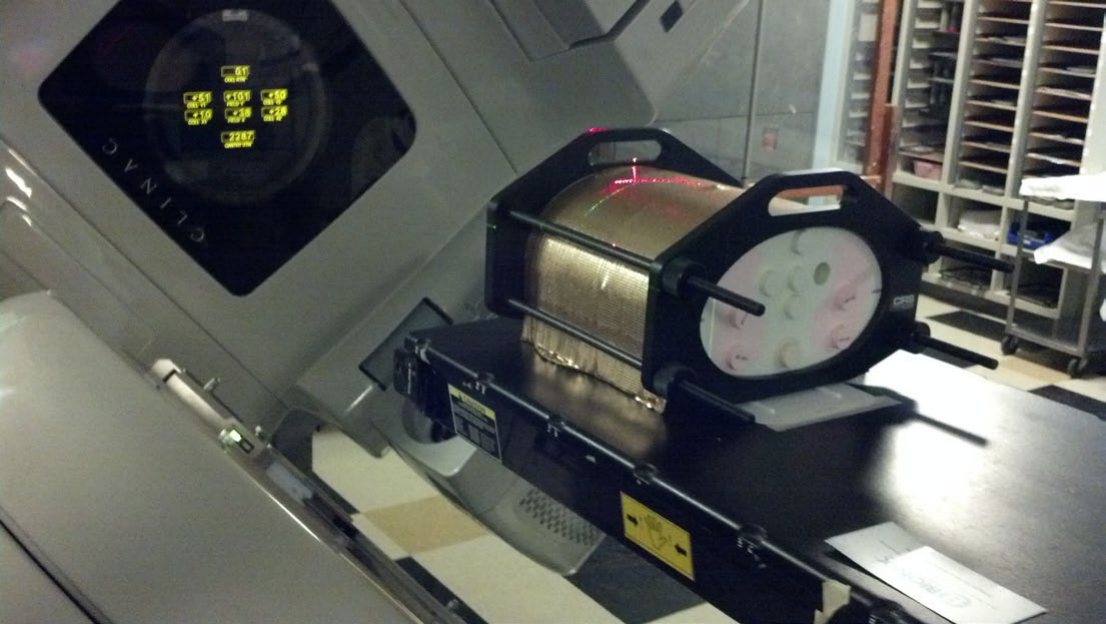
CIRS IMRT thorax phantom with brass mesh bolus.

A treatment plan was generated on a CT scan of a CIRS heterogeneous IMRT thorax phantom (CIRS, Inc., Norfolk, VA) using 6 MV photon tangent fields (Fig. 2). The treatment plan was delivered to the phantom with a layer of Gafchromic EBT3 film (Ashland, Covington, KY) taped to the surface of the irradiated area to approximate the surface dose (Fig. 3). The plan was delivered to the phantom in three different configurations: without bolus, with Superflab bolus, and with a layer of brass bolus (Fig. 4). All films used in this work were from the same batch, utilized the same calibration curve, and were scanned in the same orientation and position on the EPSON 10000XL scanner (Epson America Inc., Long Beach, CA). Surface dose was also confirmed using Landauer InLight nanoDot optically stimulated luminescent detectors (OSLDs) (Landauer, Glenwood, IL) for the brass bolus setup. For the cases utilizing bolus, every effort was made to reduce air gaps between the bolus and the phantom surface.

### D. Photoneutron production

For patients with large chest‐wall separations, sufficient dose homogeneity is difficult to achieve without delivering a portion of the treatment with higher energy photons (i.e., > 10 MV). When using brass mesh with high‐energy photon beams, the photoneutron production should be considered as activation products could present a safety hazard for the radiation oncology staff and patients. Photoneutron production in the brass mesh with higher energy beams was calculated using the Monte Carlo N‐Particle radiation transport code (MCNPX).^(9)^ The photon source characteristics were based on 15 MV and 24 MV medical linear accelerator energy and angular distributions published by Mohan et al.^(10)^ which does not include the electron component of the source term. Since the Monte Carlo model was primarily being used to determine photoneutron production, neglecting the electron component of the source term should have a minimal effect on the results. Photon transport was performed using the mcplib04^(11)^ libraries, which were derived from the ENDF/B‐VI.8 data library, based on EPDL97.^(12)^ Photoneutron production was simulated using the LA150U^(13)^ photonuclear cross section library processed by NJOY.^(14)^ Neutron transport was performed using the ENDF70 library^(15)^ which is derived from the ENDF/B‐VII.0 library.^(16)^ The photon, electron, and neutron energy cutoffs were set to 0.001 MeV, 0.1 MeV, and 0 MeV, respectively, and the integrated tiger series (ITS)‐style energy‐indexing algorithm was used to more accurately model electron transport. For each simulation, one billion source particles were transported to ensure the coefficient of variation of the metric being tallied was less than 1%.

To validate the source model, a $15\;\times \;15\;{\text{cm}}^{2}$ photon field was projected on a cubic water phantom, and the CAX PDD was calculated using an energy deposition mesh tally. The MCNPX‐calculated CAX PDD was verified against the measured CAX PDD prior to simulating the PDD in the presence of brass mesh.

The complex construction of the brass mesh is difficult to model in MCNPX. As an approximation, the brass mesh was modeled as a continuous sheet. To determine the thickness of brass mesh required in the simulation, the PDD was calculated while iterating through various thicknesses of brass mesh until the calculated PDD was within 1% of the PDD measured with the Advanced Markus ionization chamber in the buildup region. The buildup region was chosen as the depth of comparison because PDD is more sensitive to brass bolus thickness in the buildup region than at depths great than $d_{\text{max}}$. Through this process, the thickness of brass mesh was determined to be 0.4 mm (mass density of $8.55\;g/{\text{cm}}^{3}$). The physical thickness of brass mesh measured with a digital caliper was 1 mm, but a substantial portion of that 1 mm is air between the top and bottom of the brass rivet. In addition, there are small air pockets between each brass rivet. Considering this, the 0.4 mm thickness used in the MCNPX simulations is reasonable.

After determining the thickness of the brass required for MCNPX simulation, the PDD was calculated for a 15 MV and 24 MV beam in the presence and absence of photoneutron production. The resulting PDDs were compared to determine the contribution of neutrons to dose at the CAX.

Lastly, the energy‐binned neutron flux of neutrons born in the brass mesh was calculated for the cases of 15 MV and 24 MV photons impinging on the brass mesh‐covered water phantom. The ICRP 103 dose conversion coefficients^(17)^ were applied to the neutron flux distributions to estimate the effective dose per 100 monitor units (MU) for a $15\;\times \;15\;{\text{cm}}^{2}$ photon field—assuming calibration conditions of 1 cGy/MU for a $10\;\times \;10\;{\text{cm}}^{2}$ field at $d_{\text{max}}$ at 100 SAD.

### E. Activation products

As discussed in the report of the American Association of Physicists in Medicine (AAPM) Task Group 136^(18)^ photoneutrons produced by high‐energy photon beams will induce radioactivity in the linear accelerator, patient support system, building materials, the patient, and the air. The same holds true for brass mesh. Yellow brass is an alloy composed of approximately 60% copper and 40% zinc, which are two elements with nuclides having considerable thermal neutron capture cross sections. Howell et al.^(19)^ determined the photoneutron spectra and thermal neutron flux for a Varian 21EX at various nominal energies. The NIST Center for Neutron Research hosts a neutron activation and scattering calculator that will calculate the activity of activation products given some thermal neutron flux.^(20)^ This calculator was used to approximate the activity induced in the brass mesh based on the thermal neutron flux calculated by Howell et al. for a 15 MV X‐ray source. The thermal neutron flux due to neutrons born in the brass mesh was negligible; hence, they were not included in the calculation.

The next step was to determine the effective half‐life and approximate the dose rate on contact. The brass mesh was placed at isocenter (100 cm SSD) on top of a 15 cm thick slab phantom of plastic water (phantom dimensions $30\;\times \;30\;\times \;15\;{\text{cm}}^{2}$) set on the treatment table of a Varian TrueBeam linear accelerator. The gantry and collimator angles were set to 0°, the field size was set to $15\;\times \;15\;{\text{cm}}^{2}$, and a 500 MU irradiation of 15 MV X‐rays was delivered — calibration conditions of 1 cGy/MU for a $10\;\times \;10\;{\text{cm}}^{2}$ field at $d_{\text{max}}$ at 100 SAD. Following irradiation, the brass mesh was moved approximately five meters away from the gantry. A Black Cat Geiger Counter (Black Cat Systems, Westminster, MD) was used to record the change in the count rate over a 30‐minute time period, and the initial contact dose rate was estimated with a Fluke 451P Ion Chamber Survey Meter (Fluke Biomedical, Everett, WA).

## III. RESULTS AND DISCUSSION

### A. Percent depth dose (PDD)

**Figure 5:**
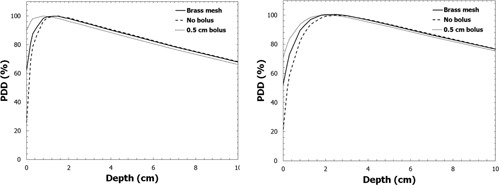
PDD curves for 6 MV (left) and 15 MV (right) beams measured with the Advanced Markus ionization chamber for the cases of 0.5 cm of tissue‐equivalent bolus, brass bolus, and no bolus.

The PDD curves of a 6 MV and a 15 MV beam measured with the Advanced Markus ionization chamber comparing the cases of 0.5 cm tissue‐equivalent bolus, one layer brass mesh bolus, and no bolus are presented in Fig. 5. For the 6 MV beam, the measured surface PDD was 25% for no bolus, 63% for one layer of brass bolus and 90% for 0.5 cm of Superflab bolus. The surface dose increase for an en face 6 MV photon beam from tissue‐equivalent bolus is substantially greater than the increase for brass mesh bolus. The PDD at 10 cm depth (100 cm SSD) was 68.2% for no bolus, 68.0% for brass mesh bolus, and 66.2% for tissue‐equivalent bolus. The dose at 10 cm depth is nearly the same for brass mesh bolus versus no bolus, while it is decreased by approximately 2% for tissue equivalent bolus. It is hypothesized that the brass mesh bolus does not shift the PDD as much as 0.5 cm of tissue‐equivalent bolus because it has a smaller tissue‐equivalent thickness (2 mm tissue‐equivalent thickness according to the manufacturer) and the beam is also hardened by the brass material.

### B. Dose‐at‐depth with tangential beams

When compared to the case of no bolus, a 0.5 cm layer of tissue‐equivalent bolus decreased the dose at a depth of 10 cm (100 cm SAD) by 1.8% for the en face beam and 2.6% for the 45° oblique beam. The brass mesh bolus decreased the dose at a depth of 10 cm by 0.6% for the en face beam and 0.9% for the 45° oblique beam. Since the dose at depth changes less than 1% in the presence of a single layer of brass bolus, a no‐bolus plan may be used for the brass bolus and nonbolus fractions.

### C. Surface dose on a thorax phantom with tangential beams

**Figure 6:**
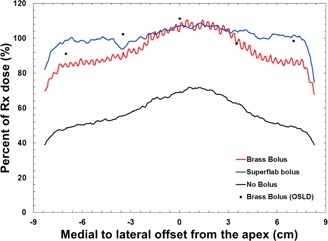
Surface dose profiles from medial (negative) to lateral (positive), using 6 MV tangential beams. Solid lines were measured with Gafchromic EBT3 film and the black dots were measured with OSLDs.

The surface dose profiles measured with Gafchromic EBT3 film and point doses measured with OSLDs when the thorax phantom was irradiated with 6 MV tangential beams are presented in Fig. 6. In the no‐bolus case, the surface dose ranged from 40%–72% of the prescription dose, with the maximum value occurring at the point where the beam entry was most shallow. The brass‐bolus surface dose profile resembles the profile for the no‐bolus case, except the surface dose is increased to 75%–110% of prescription dose. A detailed uncertainty analysis was not conducted for this work since we were not characterizing the measurement device, but these data can be assumed to have a dosimetric uncertainty within 5%. The oscillations seen in the profile are expected because the riveted chain mail construction results in inhomogeneous attenuation. The increase in surface dose measured on the IMRT thorax phantom is similar to the *in vivo* measurements of Healy et al.^(7)^ — 81%–122% in the Healy study compared to 75%–110% in this work. The surface dose under tissue‐equivalent bolus was increased to 85%–109% of prescription dose. The tissue‐equivalent bolus produces a flatter surface‐dose profile with a higher average surface dose over the medial to lateral extent of the breast. The surface‐dose profile for the tissue‐equivalent bolus case was acquired using a plan that was optimized in the presence of 0.5 cm of virtual bolus.

### D. Photoneutron production

**Figure 7:**
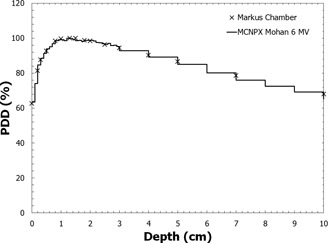
A comparison of the measured and MCNPX‐calculated PDD curves in water with brass mesh bolus for an en face 6 MV $15\;\times \;15\;{\text{cm}}^{2}$ field at 100 cm SSD.

The measured and MCNPX‐calculated PDD curves used to validate the correct thickness of brass mesh bolus to use for Monte Carlo simulation are displayed in Fig. 7. After validating the correct brass mesh thickness, the PDDs were computed for a 15 MV beam and a 24 MV beam incident on the brass‐mesh‐covered water phantom. The PDDs with and without photoneutron production were found to be within 1% over all depths, and, hence, neutron dose at the central axis in the presence of brass mesh is negligible.

### E. Activation products

**Figure 8:**
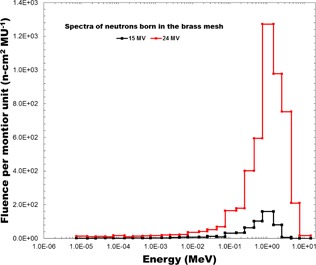
The photoneutron spectra of neutrons born in the brass mesh for 15 MV and 24 MV beams.

For the 15 MV and 24 MV Monte Carlo simulations, the photoneutron fluence spectra of neutrons born in the brass mesh were also measured, and the resulting spectra are presented in Fig. 8. To determine the increase in effective dose from the neutrons born in the mesh per monitor unit, the ICRP 103 dose conversion coefficients for an isotropic exposure to neutrons were combined with the computed photoneutron energy spectra presented in Fig. 8. The increase in effective dose from neutrons born in the brass mesh for a 100 MU en face delivery was calculated to be 1.57 μSv for 15 MV X‐rays and 17.5 μSv for 24 MV X‐rays. Assuming 300 MU per fraction and 25 fractions, the increase in effective dose over the course of treatment is 1.175 mSv for 15 MV X‐rays and 13.125 mSv for 24 MV X‐rays. As expected, the effective dose increases substantially with X‐ray energy due to the equally substantial increase in photoneutron production.

**Figure 9:**
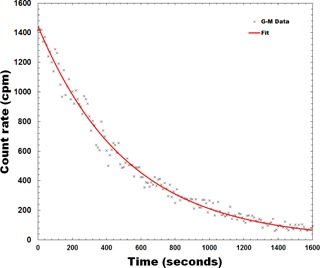
Background‐subtracted G‐M counter data representing the radioactive decay of the activation products in brass mesh bolus. The effective half‐life was calculated to be approximately 6 min.

Based on data from Howell et al.,^(19)^ the thermal neutron fluence per Gy for a Varian 21EX linac in a room with a surface area of 210 m^2^ is approximately $1\times 10^{5}\text{neutrons}/{\text{cm}}^{2}$ for 15 MV photons. The activation products and their corresponding activities of a brass mesh exposed to 500 MU of 15 MV X‐rays at a dose rate of 600 MU/min are presented in Table 1. The two copper radionuclides ${\;}^{64}\text{Cu}$ and ${\;}^{66}\text{Cu}$ are the prominent activation products due to their larger radiative capture cross‐sections. Following the 500 MU irradiation, the contact dose rate from the activation products was measured to be 0.4 mrem/hr (using a Fluke 451 survey meter). The count rate over time measured using a Geiger counter is presented in Fig. 9. The effective half‐life of the activation products was estimated to be approximately 6 min, based on a least‐squares fit to an exponential. This agrees with the expected half‐life considering the relative abundance of the activation products and their half‐lives in Table 1.

**Table 1:** Activation products produced in brass mesh bolus for 500 MU irradiation at a dose rate of 600 MU/min

*Parent*	*Rx*	*Daughter*	$t_{1/2}$	*Activity (Bq)*
${\;}^{63}\text{Cu}$	(n,γ)	${\;}^{64}\text{Cu}$	12.7 h	253.1
${\;}^{65}\text{Cu}$	(n,γ)	${\;}^{66}\text{Cu}$	5.1 m	7,656
${\;}^{64}\text{Zn}$	(n,γ)	${\;}^{66}\text{Zn}$	243.9 d	0.061
${\;}^{68}\text{Zn}$	(n,γ)	${\;}^{69}\text{Zn}$	57 m	9.449

### IV. CONCLUSIONS

Brass mesh bolus is a reasonable alternative to tissue‐equivalent bolus to increase the surface dose for a photon tangent field arrangement to treat the chest wall in PMRT. The increase in surface dose for brass bolus was comparable to that of tissue‐equivalent bolus with the exception of a slightly reduced surface dose near the medial and lateral edges of the irradiated area. The effect of the brass mesh bolus on the dose at depth is less than 1%, so one plan should be sufficient for both the brass‐bolus and nonbolus cases. The improved conformity of the brass mesh should result in less uncertainty in the surface dose for an uneven chest wall compared to tissue‐equivalent bolus. If considering the use of higher energy photon beams to improve dose homogeneity, the increased neutron dose from neutrons born in the brass mesh is substantial enough to be considered in the decision‐making process. In particular, the age of the patient should factor into the assessment of risk for secondary cancers caused by increased neutron dose. Due to the modest increase in neutron dose when using higher energy beams and in keeping with ALARA, it would be recommended to remove the brass bolus prior to delivering higher energy photon beams.

## ACKNOWLEDGMENTS

The authors would like to thank Dima Soultan for providing the Geiger counter and assisting with the Geiger counter measurements.

## COPYRIGHT

This work is licensed under a Creative Commons Attribution 3.0 Unported License.
